# Neural network model assisted Fourier ptychography with Zernike aberration recovery and total variation constraint

**DOI:** 10.1117/1.JBO.26.3.036502

**Published:** 2021-03-25

**Authors:** Yongbing Zhang, Yangzhe Liu, Shaowei Jiang, Krishna Dixit, Pengming Song, Xinfeng Zhang, Xiangyang Ji, Xiu Li

**Affiliations:** a Tsinghua University, Shenzhen International Graduate School, Department of Automation, Shenzhen, China; b Harbin Institute of Technology (Shenzhen), School of Computer of Science and Technology, Shenzhen, China; c University of Connecticut, Department of Biomedical Engineering, Storrs, Connecticut, United States; d University of Connecticut, Department of Electrical and Computer Engineering, Storrs, Connecticut, United States; e University of the Chinese Academy of Sciences, School of Computer Science and Technology, Beijing, China; f Tsinghua University, Tsinghua National Laboratory for Information Science and Technology, Department of Automation, Beijing, China

**Keywords:** Fourier ptychographic microscopy, pupil recovery, neural network, optics

## Abstract

**Significance:** Fourier ptychography (FP) is a computational imaging approach that achieves high-resolution reconstruction. Inspired by neural networks, many deep-learning-based methods are proposed to solve FP problems. However, the performance of FP still suffers from optical aberration, which needs to be considered.

**Aim:** We present a neural network model for FP reconstructions that can make proper estimation toward aberration and achieve artifact-free reconstruction.

**Approach:** Inspired by the iterative reconstruction of FP, we design a neural network model that mimics the forward imaging process of FP via TensorFlow. The sample and aberration are considered as learnable weights and optimized through back-propagation. Especially, we employ the Zernike terms instead of aberration to decrease the optimization freedom of pupil recovery and perform a high-accuracy estimation. Owing to the auto-differentiation capabilities of the neural network, we additionally utilize total variation regularization to improve the visual quality.

**Results:** We validate the performance of the reported method via both simulation and experiment. Our method exhibits higher robustness against sophisticated optical aberrations and achieves better image quality by reducing artifacts.

**Conclusions:** The forward neural network model can jointly recover the high-resolution sample and optical aberration in iterative FP reconstruction. We hope our method that can provide a neural-network perspective to solve iterative-based coherent or incoherent imaging problems.

## 1 Introduction

In biomedical applications, it is desirable to obtain complex images with both high resolution and wide field. Regardless of advancements in sophisticated mechanical scanning microscope systems and lensless microscopy setups, the modification of conventional microscopes to obtain ideal high-resolution results has been a hot topic of recent research work. Inspired by ptychography,^1^^,^^2^ Fourier ptychography (FP) in particular is a simple and cost-effective analytical method for this application.^3^^–^^7^ As a newly proposed computational imaging method, FP integrates principles of phase retrieval^8^^,^^9^ and aperture synthesizing^10^^,^^11^ to achieve both high resolution and large field of view (FOV). By introducing a programmable light-emitting diode (LED) array as an angle-varied coherent illumination source, higher-frequency information is shifted into the passband of the objective lens and detected by the image sensor. Then FP can stitch these images together in the Fourier domain to enlarge the system bandwidth via aperture synthesizing. The lost phase information is further recovered via the phase retrieval technique using intensity-only measurements. As such, FP can reconstruct a sample with both high-equivalent numerical aperture (NA) and large FOV. So far, FP has received increasing attention over the past few years, and many types of research have been proposed on the system setup and reconstruction algorithm to correct various system aberrations,^12^^,^^13^ provide robust optimization methods against noise,^14^^,^^15^ report innovative system setup designs,^16^^,^^17^ and so on.^18^^–^^20^

Recently, convolutional neural networks (CNNs) have been proven to reliably provide inductive answers to the inverse problem in computational imaging,^11^ and many biomedical imaging problems have been solved with CNN, such as computed tomography,^21^ image super-resolution,^22^ and holography.^11^^,^^23^ The purpose of FP is to converge a high-resolution target from the acquired image sequence. It is natural to introduce the idea of CNN into this inverse problem and learn an underlying mapping from the low-resolution input to a high-resolution output.^24^^–^^27^ However, there exist some specific issues in biomedical applications. First, different from natural image applications, it is hard for biomedical imaging problems to access large amounts of images. Therefore, supervised CNNs, which rely on large datasets, have encountered obstacles in training data. Second, computational imaging methods like FP are sensitive to system parameters. Once the system setup is changed or the system aberration is introduced, the performance of the deep-learning-based network will be degraded. Toward the first dilemma, Zhang et al.^27^ generated datasets with simulations to train the network. However, there is a lack of related networks to solve the second issue. To address these dilemmas properly, it is effective to add constraints based on the application characteristics. Jiang et al.^28^ proposed a physics-based framework that they established using a forward imaging model of FP via TensorFlow and utilized the back-propagation to optimize the high-resolution sample. This method uses the imaging model of FP as the constraint and solves the above two issues. The neural network is only utilized to imitate the traditional iterative recovery process. Following this idea, related methods can work with limited data^29^^,^^30^ and can work stably toward system aberration.^31^^–^^34^ However, these works are still essentially the iterative-based algorithm, and the automatic differentiation (AD) property of the neural network is not fully utilized. It would be much more desirable to design a new neural network to further degrade the noise in the reconstruction and estimate the optical aberration with higher accuracy.

In this paper, we report a neural network model to solve Fourier ptychographic problems. The Zernike aberration recovery and total variation (TV) constraint are introduced as the augmenting modules to ensure aberration corrected reconstruction and robustness against noise. As such, we name this model the integrated neural network model (INNM). INNM is essentially a TensorFlow-based trainable network mimicking the iterative reconstruction of FP. We model the forward imaging process of FP via TensorFlow at first. To estimate the optical aberration properly, the optical aberration of the employed objective lens is modeled as a pupil function in our model and optimized along with the sample through backpropagation. Then we introduce the alternate updating (AU) mechanism to achieve better performance and use the Zernike mode to make a robust and proper aberration estimation. As such, our method can recover the optical aberration with high accuracy and achieve better performance simultaneously. To further eliminate the noise, we incorporated the TV on both the amplitude and phase of the sample image. It turns out that this application can improve the image quality. Our experiments demonstrate that INNM outperforms other methods with higher contrast, especially when validated in a severe aberration condition.

This paper is structured as follows. In Sec. 2, we discuss the fundamental principles and reconstruction procedure of FP. In Sec. 3, we describe the model structure and introduced mechanisms step by step. Then in Sec. 4, we validate our method on both simulated and experimental datasets under variant acquisition conditions and analyze the benefits of introduced methods in detail. Finally, we provide concluding remarks and discuss our ongoing efforts in Sec. 5.

## 2 Fourier Ptychographic Microscopy

**Fig. 1:**
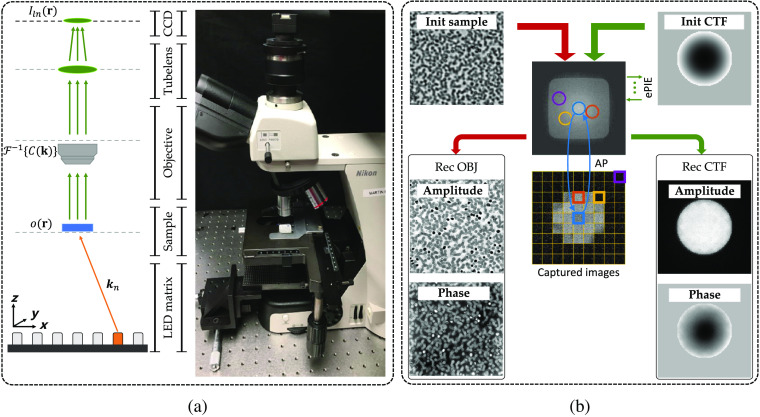
Fundamental principles of FP: (a) the schematic diagram of the FP experimental setup and the physical comparison and (b) the iterative decomposition procedure with pupil recovery.

As a classic analytical method, FP is mainly composed of the explicit forward imaging model and the decomposition procedure. Considering the generalized FP schematic diagram setup shown in Fig. 1(a), the sample is successively illuminated by plane waves from the LED matrix at different angles. The exit waves are then captured by the image sensor (CCD) through the objective lens. Sequentially lighting distinct LEDs on the matrix, FP can obtain a low-resolution intensity image sequence to recover a high-resolution complex one.

The forward imaging procedure is shown in the left column of Fig. 1(a). We denote the thin sample as its transmission function o(r), where r=(x,y) represents the 2D spatial coordinates with its Fourier expression as $k=(k_{x},k_{y})$. When obliquely illuminated by the n’th monochromatic LED, the exit wave at object plane can be denoted as $o(r)⊙\exp(ik_{n})$, where ⊙ denotes the element-wise multiplication and $k_{n}=(k_{x}^{n},k_{y}^{n})$ denotes the n’th wave vector corresponding to the angle of incident illumination. The final wave captured by the CCD can be expressed as

$$I_{\ln}(r)={\left|\{o(r)⊙\exp(ik_{n})\}*F^{-1}\{C(k)\}\right|}^{2},$$ (1)

where F denotes Fourier transformation, * denotes convolution, C(k) denotes the coherent transfer function (CTF) of the objective lens, and $I_{\ln}(r)$ denotes the captured image under n’th LED illumination. Here we note that subscripts l, h, and n denote low resolution, high resolution, and sequence number, respectively. The standard formulation of the pupil function CTF can be expressed as

$$C(k)=\{\begin{array}{ll} 1, & (k_{x}^{2}+k_{y}^{2})<{\left(\mathrm{NA}\cdot k_{0}\right)}^{2},\\ 0, & \text{otherwise}, \end{array}$$ (2)

where NA characterizes the range of angles over which the system can accept light, $k_{0}=2\pi /\lambda$ and λ is the illumination wavelength.

As for the decomposition procedure shown in Fig. 1(b), FP will first initialize a high-resolution sample image. Then FP will shift the confined CTF into distinct apertures and utilize a phase retrieval method called alternating projection (AP) to obtain a self-consistent complex image. In AP, the pinned aperture in the Fourier domain will be updated by keeping its spatial phase unchanged and replacing its spatial amplitude with the square root of the corresponding captured image. In addition, since optical aberration is a common issue in practical applications,^35^ it is valuable to embed an extra pupil recovery called ePIE^4^ after AP to compensate for this interference. The whole decomposition process with pupil recovery is shown in Fig. 1(b) (the red flowchart denotes the original FP and the green one denotes the extended pupil recovery).

## 3 Method

### 3.1 Framework of INNM

**Fig. 2:**
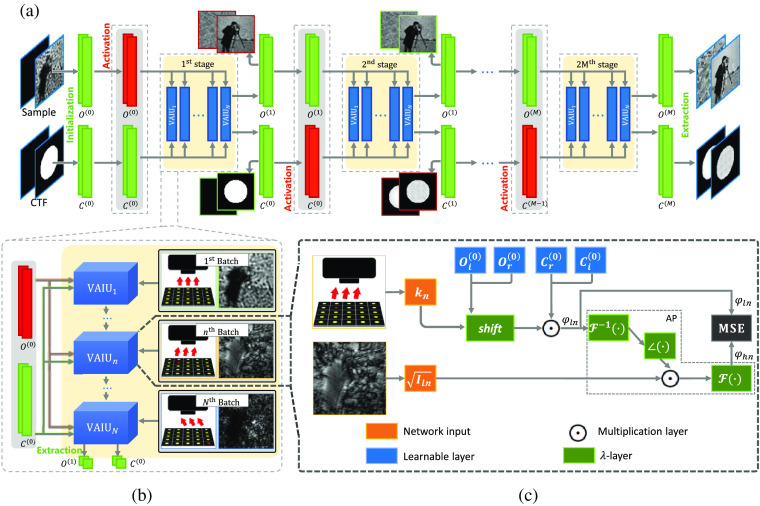
Illustration of the INNM framework: (a) schematics of pipeline procedure with the AU mechanism; (b) schematics of a training epoch; and (c) the basic framework of INNM with embedded pupil recovery.

To solve FP problems via the neural network, we model the forward imaging process via TensorFlow, which could obtain the sample and optical aberration simultaneously. Figure 2 illustrates the detailed pipeline workflow and the framework of our method. As shown in Fig. 2(a), the upsampled central measurement and the standard CTF without pupil aberration are set to the initial guess of the sample and CTF, respectively. Then the optimized targets can be obtained through several training stages. Each training stage consists of a few epochs (for simplicity, only one epoch is shown). As shown in Fig. 2(b), we take the pair of captured image $I_{\ln}(r)$ and corresponding plane wave vector $k_{n}$ as a single batch. In each epoch, all batches are fed into the model, and the model parameters are updated through backpropagation. To avoid confusion, we termed these batches as varying angle illumination units (VAIUs) and represented them as a complete epoch.

The detailed framework of our model with the embedded pupil recovery is shown in Fig. 2(c). This model is transferred from the forward imaging process in the Fourier domain, and the whole process can be formulated as follows:

$$\varphi_{\ln}(k)=O(k+k_{n})⊙C(k),$$ (3)

$$\varphi_{hn}(k)=F\{\sqrt{I_{\ln}(r)}⊙∠\{F^{-1}\{\varphi_{\ln}(k)\}\}\},$$ (4)

where O(k) denotes the Fourier function of the sample, ∠ denotes the phase, $\varphi_{\ln}$ and $\varphi_{hn}$ represent the original and updated apertures in Fourier domain during the AP.

Reflected in Fig. 2(c), the sample and CTF are naturally treated as two-channel (with the real and imaginary parts) learnable feature maps. Then the sample O(k) in the Fourier domain is forward propagated through the well-designed framework following Eq. (3), where the sample is first shifted according to $k_{n}$ and multiplied with the CTF C(k). Then the generated spectrum $\varphi_{\ln}(k)$ is transmitted via the AP defined in Eq. (4) with the preupsampled input $I_{\ln}(r)$. Thus we can generate the updated spectrum $\varphi_{hn}(k)$. Based on the phase retrieval mechanism, these two exit wave series of spectra $\varphi_{\ln}(k)$ and $\varphi_{hn}(k)$ should contain the same frequency information when converged. Therefore, the whole framework can obtain optimized results by minimizing the differences between these spectra. The first loss function used in INNM can be expressed as

$$\text{loss}=\Sigma_{n=0}^{N}{\left\|\varphi_{hn}(k)-\varphi_{\ln}(k)\right\|}_{2}^{2},$$ (5)

where L2-norm is used to measure the differences and N denotes the number of VAIUs.

As for the detailed composition of the network, self-designed fixed -layers and multiplication layers are widely used to perform operations such as AP. More specifically, the -layer can be flexibly customized to define operations, such as using the roll function in TensorFlow to perform the shifting operation. As such, these layers can be designed to perform the forward imaging model of FP in TensorFlow. We should note that INNM is defined in the complex field, and all the layers consist of the real and imaginary parts. For example, we should rewrite Eq. (3) in the complex domain as

$$\varphi_{\ln}(k)=\{O_{r}(k+k_{n})+i\cdot O_{i}(k+k_{n})\}⊙\{C_{r}(k)+i\cdot C_{i}(k)\}\;=(O_{r}⊙C_{r}-O_{i}⊙C_{i})+i\cdot (O_{r}⊙C_{i}+O_{i}⊙C_{r}),$$ (6)

where the subscripts r and i denote the real and imaginary parts, respectively. At last, customized network layers are used to represent the learnable targets, like sample and CTF. As such, the model is lightweight with limited trainable parameters (sample and CTF). After backpropagation, we can extract parameters in the learnable layers shown in the blue boxes of Fig. 2(c) to get the optimized results. Our Jupyter notebook source code for this algorithm has been made available; a link is provided in the Data, Materials, and Code Availability section, below.

### 3.2 Alternate Updating Mechanism

Based on the model designed in Fig. 2, we can directly obtain the recovered sample and estimated CTF in theory. However, the sample and CTF have different sensitivities toward the backward gradients. The model cannot converge to the optimal result if the sample and CTF are updated simultaneously with the same step size of gradient descent. As such, the AU mechanism is devised to control the optimized target and the step size of gradient descent.

In general networks, the training process is always consecutive. Here we divide the training process of INNM into plural stages, and the updating objectives differ in adjacent stages. The approach of how we control the updated parameters has shown in Fig. 2(a). In the first stage, we believe the CTF is closer to the practical ground truth. Then the parameters of CTF $C^{\left(0\right)}$ will be fixed, and only the sample parameter $O^{\left(0\right)}$ can be updated by backpropagation. Then we will update the CTF $C^{\left(0\right)}$ and keep $O^{\left(1\right)}$ unchanged since the updated sample $O^{\left(1\right)}$ contains more detailed and realistic information. In short, INNM alternately keeps either the sample or the CTF constant in a stage. Then INNM can assign the sample and CTF individual learning rates to control the step size of gradient descent. The targets can be better optimized. As the model parameters are alternately updated, the sample and CTF will gradually converge to the optimal point.

For clarity, the details of the sample and CTF in distinct stages are provided in Fig. 2(a). In the first stage, the amplitude and phase images of the sample are improved, and in the second stage, the CTF is updated. We can obtain the estimated CTF and the recovered aberration-free object in the last stage. As such, we can demonstrate that the benefit of applying AU into the INNM is that this mechanism can help to update the parameters more appropriately by controlling the optimized target.^36^

### 3.3 Improved Modality of Optical Aberration

In FP applications, if the sampling pattern is a periodic grid in the Fourier domain, it would introduce periodic artifacts to the recovered pupil function. Then this corrupted pupil function will degrade the high-resolution FP reconstruction.^18^ To address this problem, the Zernike polynomials are incorporated due to their powerful ability to describe wavefront characteristics in optical imaging.^37^^–^^39^

In addition, the CTF is always updated as a whole. Nevertheless, if the Zernike mode is applied to model the CTF, the parameters that need to be compensated for the aberration are reduced from the square of image size to the constant number of Zernike modes that need to be fitted. In other words, the optimization degrees of freedom are orders of magnitude lower than the previous implementations.

As such, the new modality of CTF’s phase is updated as

$$∠C(k)=\overset{L}{\underset{l=1}{\sum }}c_{l}\cdot Z_{l}(k),$$ (7)

where L denotes the number of Zernike modes used in the model, and $c_{l}\in R$ is the coefficient of each polynomial $Z_{l}(k)$. In general, the first nine modes after piston $Z_{0}^{0}$ can already fit the common aberration in microscopy. Consequently, we can only train nine parameters to achieve the same performance as that of its entirety. As for the CTF’s amplitude, it will still be updated as a whole. As such, C(k)=|C(k)|⊙exp{i∠C(k)} is the final form we used in INNM to model the CTF.

### 3.4 Enhanced Loss Function

One of the strengths of neural networks lies in the auto-differentiation capabilities of optimization toolboxes. Therefore, different cost functions can lead to different results. If the appropriate functions are introduced, the model can achieve better results.

In FP applications, the recovered sample image will contain noise due to the limited synthesized NA. As such, considering reduce noise without degrading edges, we can use the TV regularizer to achieve better performance. TV regularization is widely used in the field of signal processing and so on.^40^^,^^41^ By calculating the integral of the absolute gradient, it evaluates the degree in which the image is disturbed by noise.

We expand the loss function Eq. (5) with TV as follows:

$$\text{loss}={\left\|\varphi_{hn}(k)-\varphi_{\ln}(k)\right\|}_{2}^{2}+\alpha_{1}\cdot \mathrm{TV}\{|\Phi_{hn}(r)|\}+\alpha_{2}\cdot \mathrm{TV}\{∠\Phi_{hn}(r)\},$$ (8)

$$\mathrm{TV}\{o(r)\}=\sum {\left({\left|o_{x+1,y}-o_{x,y}\right|}^{2}+{\left|o_{x,y+1}-o_{x,y}\right|}^{2}\right)}^{\eta /2},$$ (9)

where |·| and ∠ represent the amplitude and phase of the complex sample $\Phi_{hn}(r)$. In addition, $\Phi_{hn}(r)$ is the transmission function of the spectrum $\varphi_{hn}(k)$ in the spatial domain. Equation (8) is composed of three parts, where ${\left\|\varphi_{hn}(k)-\varphi_{\ln}(k)\right\|}_{2}^{2}$ denotes the L2-norm during the AP, $\mathrm{TV}\{|\Phi_{hn}(r)|\}$ and $\mathrm{TV}\{∠\Phi_{hn}(r)\}$ represent the TV regularization of the amplitude and the phase of the updated image. Parameters $\alpha_{1}$ and $\alpha_{2}$ denote the coefficients of two TV terms, respectively. The larger the values are, the smoother the reconstructed images are. The standard formulation of the TV function is expressed as Eq. (9), where o(r) is used as a template, η represents the power series of TV, and η=1 is used in our experiments. Since the TV regularization is applied to improve the spatial smoothness of two relevant targets, our method can adjust the performance by choosing different TV coefficient combinations.

### 3.5 FP Reconstruction Procedure with INNM

Combining the above mechanisms, our model can effectively reconstruct the sample and CTF with smooth background and high image contrast.

As shown in Fig. 2(a), the whole procedure can be divided into three parts. In the initialization stage, the upsampled intensity image and the standard CTF without pupil aberration are used as the initial guesses. Next, the sample and CTF are modeled as learnable weights of hidden layers according to Eqs. (3), (4), and (7) and optimized according to Eq. (8). The gradient is then calculated by the auto-differentiation of TensorFlow to optimize the learnable parameters. It should be noted that the captured images should be preupsampled to satisfy the size requirements. In the output stage, we can obtain the sample with recovered CTF simultaneously by extracting the optimized weights of hidden layers.

## 4 Experiments

We validated our INNM on both simulated and experimental datasets. For comparison, we mostly compared our results with the widely used method ePIE,^4^ which is a robust and effective FP method with pupil recovery. We also compared with other methods over the experimental dataset, such as Jiang’s method^28^ and AS.^42^

### 4.1 System Setup

The whole setup with its schematic diagram is shown in Fig. 1(a). In our setup, a programmable 15×15 LED matrix (532 nm central wavelength) is placed ∼90  mm below the sample plane for illumination. The distance between adjacent LEDs is 4 mm. Then the 2×, 0.1 NA objective lens with a 200-mm tube lens is used to build the FP microscopy system, and a camera with a pixel size of 3.45  μm is utilized to capture low-resolution intensity images. As such, the overlap ratio in the Fourier space is about 78% and the synthetic ${\mathrm{NA}}_{\mathrm{syn}}$ could be up to 0.50.

For the generation of simulated datasets, we chose two images with a size of 128×128 as the ground truth. Then 225 32×32 simulated intensity images were generated based on the above system setup. To be more authentic, we additionally assumed the simulated images were captured with a defocus aberration, where the defocus mode $Z_{2}^{0}$ is about -1.44, corresponding to 50  μm defocus.

For the acquisition of experimental datasets, we captured the images under two different conditions. First, we obtained two tissue section datasets with the image size of 64×64. The samples were well placed on the focal plane, and the aberration could be ignored. Then we designed an extreme but valuable extension that we crop a 128×128 tile close to the edge of the entire FOV with severe aberration.

For the experimental datasets, we alternately updated the sample and CTF with 10 stages and 5 epochs for each stage. For simulated datasets, such hyperparameters would be different since the image size was smaller.

### 4.2 Results on Simulated Datasets

**Fig. 3:**
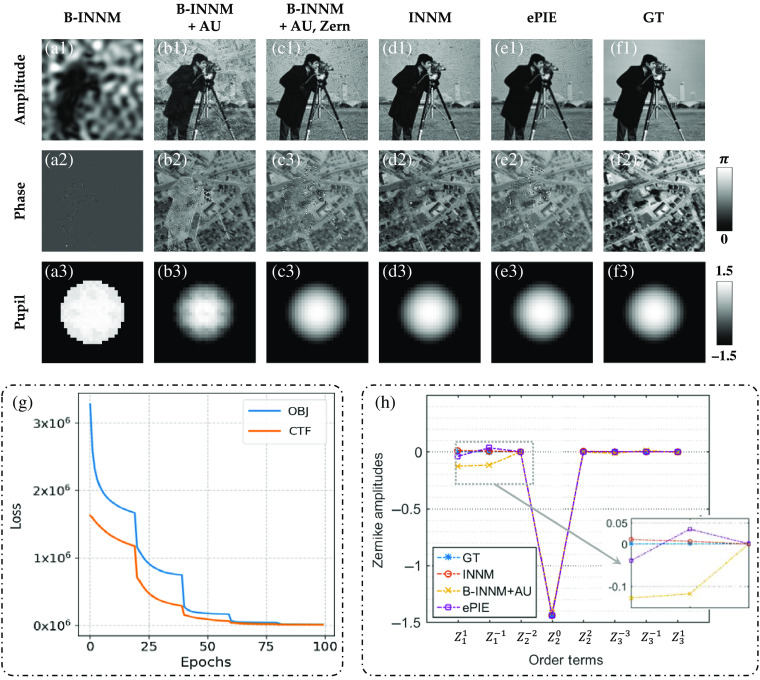
Comparison of recovered results and some decomposed Zernike amplitudes. (a)–(f) Recovered results of the sample and pupil function under different methods (the amplitude are normalized into 0 to 1). (g) Decline curve of the INNM. (h) A scatter plot of some decomposed Zernike polynomial coefficients $c_{l}$ (piston coefficient $Z_{0}^{0}$ is not present).

The ground truth of the simulated dataset is shown in Fig. 3(f). The cameraman and street map are used as the amplitude and phase images. The defocus aberration is set to be the optical aberration. The goal of reconstructed methods is to extract the sample and CTF from 225 32×32 intensity images.

#### 4.2.1 Alternate updating process

For clarity, we call the framework of INNM in Sec. 3.1 as B-INNM. Then recovered results under different settings of INNM are represented in Figs. 3(a)–3(d), and INNM stands for B-INNM with all the attributes. Considering the influence of the AU, we compare the recovered results shown in Figs. 3(a) and 3(b). It is apparent that the former sample images are quite ambiguous, and the estimated aberration is also meaningless. Instead, the latter one contains fewer artifacts and achieves higher fidelity, owing to the introduction of the AU mechanism. In addition, we represent the training curve with AU shown in Fig. 3(g). Based on the above discussion, we can demonstrate that AU can lead to the expected optimum by controlling the optimized target and compensate for the aberration to some extent. However, the recovered aberration contains unexpected periodic speckles [Fig. 3(b3)]. This problem is caused by the periodic grid sampling pattern in the Fourier domain and would degrade the high-resolution FP reconstruction.^18^ To solve this problem, additional principles are incorporated.

#### 4.2.2 Zernike polynomial function

As shown in Fig. 3(c), the greatest contribution made by the Zernike mode is the proper correction of the aberration. Compared to the aberration shown in Fig. 3(b3), the new aberration with the Zernike modes completely removes the pollution of raster-grid speckles. Furthermore, the decomposed Zernike coefficients under various conditions are illustrated in Fig. 3(h). We can find that INNM can fit the polynomial coefficients with high accuracy in all order terms, whereas B-INNM with AU cannot restore these parameters. As such, the biased coefficients in tilt Zernike modes ($Z_{1}^{-1}$ and $Z_{1}^{1}$) lead to the raster-grid speckles in Fig. 3(b3). When this issue is resolved, the Zernike mode helps to achieve the higher reconstruction quality shown in Fig. 3(c). Such a phenomenon proves that taking the optical aberration as a power series expansion is better for optimization than taking it as a whole. In addition, we can further find that the decomposed coefficient amplitudes obtained from ePIE are differential from the ground truth in the tilt Zernike modes. This disadvantage will hinder the high-quality reconstruction shown in Fig. 3(e).

#### 4.2.3 Total variation loss

By introducing the above two mechanisms into the B-INNM, our method can predict the optical aberration with high accuracy and eliminate its interference. Here we will provide the results of integrating the TV loss in the final objective function. In FP, the recovered amplitude and phase can always crosstalk with each other and cause background artifacts. As shown in Fig. 3(c), even though we properly estimate the aberration, the recovered sample images still suffer from this noise. To degrade unexpected noises, we introduce the TV regularization and the modified result is shown in Fig. 3(d). It is obvious that the background noise is greatly degraded and achieves higher image quality. In the end, we compare our results [Fig. 3(d)] with ePIE [Fig. 3(e)]. We can clearly find that our reconstructed phase completely eliminates the amplitude interference while ePIE cannot, owing to the introduction of advanced mechanisms.

In summary, by reproducing the imaging system through neural network tools and incorporating advanced mechanisms, our method provides a new perspective for FP reconstruction and can achieve higher image quality against optical aberrations and artifacts.

### 4.3 Results on Experimental Datasets

In this section, we implement our method over several experimental datasets captured under normal and severe conditions.

#### 4.3.1 Normal condition

**Fig. 4:**
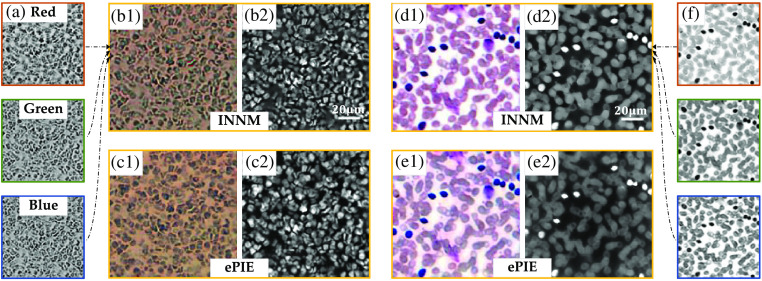
Reconstruction of two datasets in a low-aberration condition. (a) Recovered amplitudes at 632, 532, and 470 nm wavelengths from INNM. (b), (c) The combined color intensity and phase images of a tissue section stained by immunohistochemistry methodology from INNM and ePIE. (d)–(f) Recovered results of blood cells.

We first implement our framework over two general datasets where the samples are well placed on the focal plane. Figure 4 shows the reconstructions of a tissue section stained by immunohistochemistry methodology and a blood smear. In Fig. 4(a), we show the three recovered images of the tissue section illuminated by red, green, and blue monochromatic LEDs. By combining the three images together, the recovered color amplitude is shown in Fig. 4(b1) with its phase shown in Fig. 4(b2). The corresponding results from ePIE are shown in Fig. 4(c). The tissue section dataset is carefully captured so that the aberration could be ignored during the reconstruction. As such, we can find that both INNM and ePIE can obtain images with high quality.

In addition, we test the two methods in another blood smear dataset shown in Figs. 4(d)–4(f) and draw the same conclusion. The recovered sample images from ePIE and INNM share highly similarly detailed information such as cellular structures and phase distribution, and we can clearly see the performance improvement from these reconstructions compared to raw images shown in Fig. 1(b).

#### 4.3.2 Severe condition

Although the image reconstruction capabilities of INNM and ePIE are similar in generic datasets, the introduced mechanism introduced by INNM greatly enhances the potential to alleviate the optical aberration. We deliberately crop a tile close to the edge of the entire FOV, in which the system aberration is so sophisticated that seriously affects the qualities of captured intensity images. To make our results more credible, we additionally test INNM under different network settings, adjust the number of Zernike modes L to 50, and make an extra comparison with Jiang’s method^28^ and AS.^42^

**Fig. 5:**
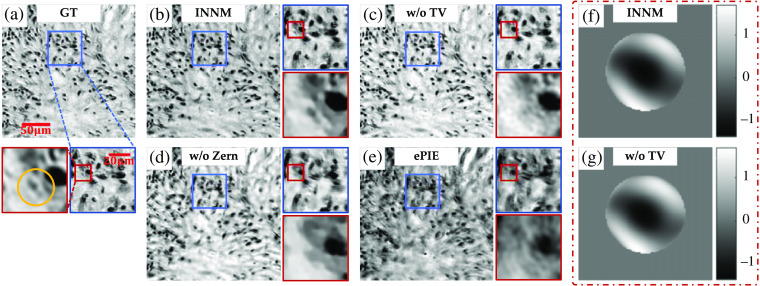
Recovered amplitude images of a tissue slide in condition of severe aberration: (a) high-resolution ground truth; (b)–(d) recovered amplitudes from INNM under different conditions; (e) recovered amplitude from ePIE; and (f), (g) aberrations restored from INNM with and without TV.

The reconstructed amplitude images with some magnified areas are shown in Fig. 5 (the ground truth is obtained from the aberration-free dataset). First, compared with the ground truth shown in Fig. 5(a), we can easily find that ePIE cannot recover the amplitude with distinguishable cell structures [Fig. 5(e)], which also happens in the reconstruction of INNM without Zernike function [Fig. 5(d)]. Then when focusing on the center of the secondary magnification area, we can find that reconstruction obtained by the INNM without TV is rough in detail due to the noise [Fig. 5(c)], and the information of small cellular structure is totally lost. In contrast, the complete INNM can distinguish this small cell characteristic from surrounding tissue structures, and the overall image is high quality with a smooth background [Fig. 5(b)].

**Fig. 6:**
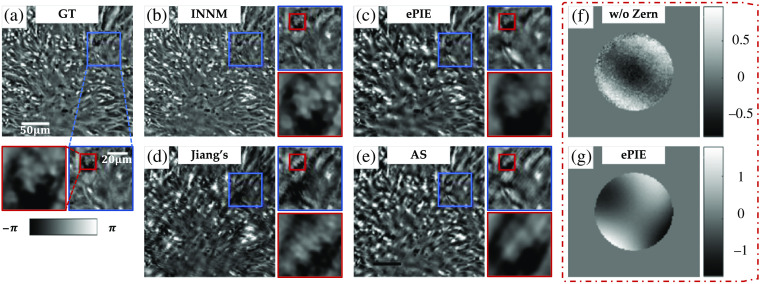
Recovered phase images in condition of severe aberration: (a) ground truth; (b)–(e) recovered phases from INNM, ePIE, Jiang’s method, and AS;^42^ and (f), (g) aberrations restored from INNM without Zernike mode and ePIE.

In Fig. 6, we further compare the phase images recovered from INNM, ePIE, Jiang’s method, and AS, in which the first two methods take optical aberration into account and the latter two do not (all the methods start with the zero-aberration hypothesis). It is clear that reconstructions from Jiang’s method and AS are highly blurred and cannot provide instructive information due to the influence of aberrations. For more recognizable details shown in the secondary magnification area, we can find that INNM achieves higher contrast than ePIE, compared with GT shown in Fig. 6(a). Also the cell structure of ePIE is quite blur compared with INNM.

Reflected in the recovered optical aberration, the results of ePIE and INNM without the Zernike mode [Figs. 6(g) and 6(f)] are quite different from those of INNM with and without TV [Figs. 5(f) and 5(g)], resulting in poor performance on recovered images shown in Fig. 5. This phenomenon further proves the superiority of INNM in compensating aberration.

In conclusion, INNM can reconstruct sample images with high quality in both simulated and experimental datasets. Especially, when validated in severe aberration conditions, INNM performs higher robustness against sophisticated optical aberrations and achieves better performance than other methods. This improved performance relies on the augmenting modules like Zernike aberration recovery on the one hand, and the AD of optimization tools in neural networks on the other hand. The AD approach allows solving FP problems without finding an analytical derivation of the update function since the derivation could be challenging to obtain. In addition, AD is directly benefited from the progress made in the machine-learning community in terms of hardware, software tools, and algorithms. We can expect INNM to be further improved thanks to the fast-paced progress in AD.

## 5 Conclusion

In this work, we reported a reconstruction method based on the neural network model to solve FP problems and achieved artifacts-free performance. By reproducing the imaging process into the neural network and modeling the sample and aberration as learnable weights of multiplication layers, INNM can obtain an aberration-free complex sample. Advanced tools are further introduced to ensure good performance. The AU can help INNM optimize the sample and aberration in an appropriate alternative way, the introduced Zernike mode can estimate the sophisticated optical aberration with high accuracy and the TV terms is useful to reduce artifacts by encouraging spatial smoothness. We tested our method over both the simulated and experimental datasets, and the results demonstrated that INNM could reconstruct images with smooth backgrounds and detailed information. We believe our recovered aberration can be used as a good estimation toward system optical transmission and we hope our method can provide a neural-network perspective to solve the iterative-based coherent or incoherent imaging problem. Moreover, we can expect such techniques can be further improved thanks to the fast-paced progress in deep-learning toolboxes like TensorFlow or powerful tools like novel regularizations and optimizers.

There are many works worth trying in the future. For example, we can replace some layers of INNM with advanced architectures so that the model can learn some advanced features in advance. In addition, although we can foresee the effect of different coefficient combinations in TV terms, manual adjustment is still required for redundant operation in practice. As such, how to optimize the image evaluation system, with which the network model can automatically adjust the performance, is also a feasible direction of research.

## Data, Materials, and Code Availability

Our code for simulation is available in the Figshare repository via the link https://figshare.com/articles/PgNN-Code-zip/11906832.
